# Development of a Portable 3CCD Camera System for Multispectral Imaging of Biological Samples

**DOI:** 10.3390/s141120262

**Published:** 2014-10-27

**Authors:** Hoyoung Lee, Soo Hyun Park, Sang Ha Noh, Jongguk Lim, Moon S. Kim

**Affiliations:** 1 Environmental Microbial and Food Safety Laboratory, Beltsville Agricultural Research Center Agricultural Research Service, United States Department of Agriculture, 10300 Baltimore Ave, Beltsville, MD 20705, USA; E-Mail: hoyoung.yi@gmail.com; 2 Department of Biosystems & Biomaterials Science and Engineering, Seoul National University, Seoul 151-921, Korea; E-Mails: ecoloves@gmail.com (S.H.P.); noh@snu.ac.kr (S.H.N.); 3 National Academy of Agricultural Science, Rural Development Administration, 150 Suinro, Gwonseon-gu, Suwon, Gyeonggi-do 441-100, Korea; E-Mail: limjg@korea.kr

**Keywords:** multispectral imaging, portable 3CCD Camera, beam splitter, prisms, parallel processing, apple defect detection

## Abstract

Recent studies have suggested the need for imaging devices capable of multispectral imaging beyond the visible region, to allow for quality and safety evaluations of agricultural commodities. Conventional multispectral imaging devices lack flexibility in spectral waveband selectivity for such applications. In this paper, a recently developed portable 3CCD camera with significant improvements over existing imaging devices is presented. A beam-splitter prism assembly for 3CCD was designed to accommodate three interference filters that can be easily changed for application-specific multispectral waveband selection in the 400 to 1000 nm region. We also designed and integrated electronic components on printed circuit boards with firmware programming, enabling parallel processing, synchronization, and independent control of the three CCD sensors, to ensure the transfer of data without significant delay or data loss due to buffering. The system can stream 30 frames (3-waveband images in each frame) per second. The potential utility of the 3CCD camera system was demonstrated in the laboratory for detecting defect spots on apples.

## 1. Introduction

To ensure high quality and safety of food products, agricultural commodities are sorted, graded, and inspected for defects in production and processing chains. Traditionally, human-based visual inspection for grading and sorting for color, size and shape, and detecting defects has played a significant role in food production. For many quality assessments of agricultural products, modernized automated food processing has replaced human labor with machine vision technologies. Research on industrial applications of machine-vision-based inspection for food quality first employed either monochromatic or RGB-based imaging technologies [1–3]. In more recent years, research and development of machine vision systems for inspecting agricultural commodities have expanded into spectral regions beyond the visible (Vis) range to include near-infrared (NIR) methods. To enhance inspection specificity, such as for defect detection on fruits, multispectral imaging approaches in the Vis/NIR have been investigated using hyperspectral imaging techniques [4–7]. In these studies, the use of two or three spectral bands could effectively detect defects presented on the surface of agricultural commodities [8–11].

For acquisition of multispectral images (excluding RGB-CCDs), several approaches have been investigated and developed: a system consisting of a interference filter wheel and a panchromatic camera to sequentially capture multispectral images [12,13], a system that uses multiple CCD cameras with internally-installed interference filters to simultaneously capture multispectral images [14,15], a system employing a common aperture-based adapter using prisms and interference filters to project 4-multispectral images to a single focal plane of CCD detector [16,17]. In recent years, line-scan (push broom) hyperspectral imaging with selection of a few spectral regions of interest has been used as a multispectral imaging platform for online agricultural commodity inspection [18,19]. This line-scan hyperspectral-multispectral method has demonstrated the added benefit of software-selectable spectral bands out of the contiguous hyperspectral bands. However, sequential acquisition of many line-scans across an object are necessary to construct images at multispectral bands; this technique is suitable for in-line agricultural commodity inspection applications as sample materials travel across the linear field of view (FOV) of the line-scan imaging system.

For rapid multispectral imaging of stationary samples in two to three spectral bands, common aperture-based CCDs, with separate individual CCDs simultaneously capturing multispectral images, are ideal. Commercially available 3CCD camera systems are configured with sealed dichroic prisms, and the spectral filtering materials are vaporized and deposited onto the prism surfaces to achieve multispectral imaging [20–22]. Such multispectral imagers lack flexibility in spectral band selection, and thus are limited to customized use in specific industrial and scientific applications.

This methodology paper presents an improved common-aperture-based 3CCD system to overcome the limitations inherent to current commercially available systems. To allow flexibility in the use of (exchangeable) interference filters for multispectral imaging, prism geometries were configured (3-channel neutral separation) with additions of user-accessible interference filter holders/housing. This design enhancement enables application-specific waveband selection for 3-channel multispectral imaging. Furthermore, we incorporated a field-programmable gate array (FPGA) module to perform parallel processing for multispectral imaging synchronization without time lags between individual image acquisitions. An additional electronic design feature includes independent control of each CCD for adjusting gain (brightness) and exposure time. The acquisition of representative multispectral images of apples with defects was demonstrated using the newly developed 3CCD system configured with 740, 820, and 1000 nm interference filters are presented.

## 2. Critical Components

**Figure 1.**
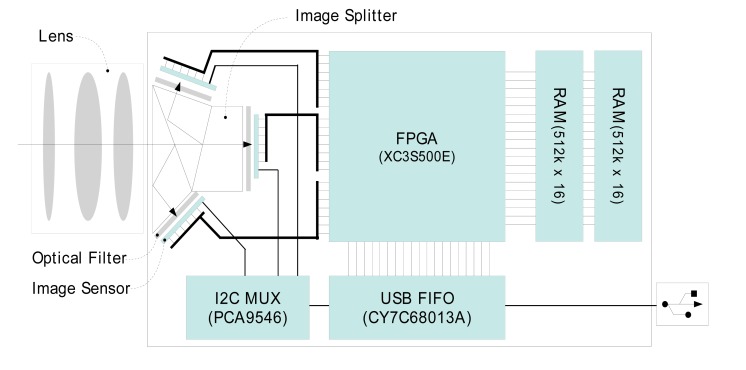
The schematic layout of critical components of the portable multispectral 3CCD camera. Design and development include in-house fabrication of the beam-splitter prism assembly, integration of the electrical components on printed circuit boards, and firmware programming to achieve parallel processing of image data from 3CCDs.

The overall layout and schematics of critical components of the 3CCD camera is shown in Figure 1. The optical components are composed of an object lens, prism-based beam (image) splitter, and interference filters. Light through the object lens is divided into three optical planes via the beam splitter, and interference filters positioned between the beam splitter and the focal planes allow multispectral imaging at three wavebands. This configuration with user-accessible interference filter holders enables the exchange of interference filters for application-specific multispectral wavebands.

The key electronic components include an FPGA (Field-Programmable Gate Array, XC3S500E, Xilinx, San Jose, CA, USA) module, two 16-bit 512K RAM (Random Access Memory, CY7C1061, Cypress, San Jose, CA, USA), USB (Universal Serial Bus) FIFO (First-In First-Out, CY7C68130A, Cypress) module, and I2C (Inter-Integrated Circuit) multiplexer (PCA9545, Texas Instruments, Dallas, TX, USA). For imaging, three 1280 × 1024 pixel, 6.66 × 5.32 mm CCD sensors (OV9121, OmniVision, Santa Clara, CA, USA) were used. For synchronization of the CCD sensors, ISP (image signal processing), and flow control of the image data, the FPGA module is programmed to function as a multifunctional controlling device. A USB FIFO module accomplishes USB 2.0 high speed communication (480 Mbits/s) with the host computer for transporting image data and controlling the sensor parameters. The RAM module is employed to temporarily buffer image data to optimize data transfer (without the loss of data) between the imaging system, USB FIFO, and the host computer.

## 3. Design and Development of the Portable 3CCD Camera

### 3.1. Beam-Splitter Prism Assembly

**Figure 2.**
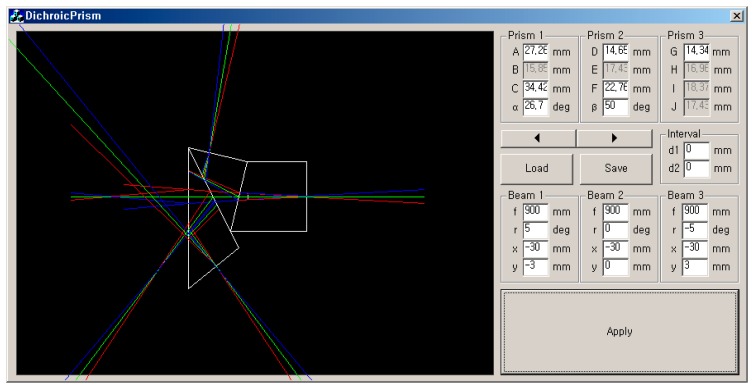
A screen capture of the simulation software. Simulation shows the wavelength-dependent path of incident rays and provides optimal arrangements (*i.e.*, angles and dimensions) of the three prisms.

The beam-splitter prism assembly was designed to accommodate the placement of interference filters instead of the typical dichroic coating used on prisms, and to further allow changing of the filters for application-specific multispectral imaging. The 3-waveband beam splitter assembly was designed with 35-mm back focal length for 6.66 × 5.32 mm CCD with consideration for spacing for the interference filter placements between the prism assembly and the CCD image sensors. The beam-splitter prisms were designed to work in the NIR portion (from 700 to 1000 nm) of the silicon-based CCD sensors. A high quality optical glass (borosilicate crown glass, BK7) was used for the beam-splitter prism assembly. In order to determine the dimensions of the three-prism assembly (with a minimum of 5 mm space between the prism assembly and the CCD sensors to accommodate the interference filter/holder assembly) and to trace the pathway of incident rays, the simulation software program shown in Figure 2 was developed in Visual C++ (Microsoft Foundation Classes, Microsoft, Redmond, WA, USA).

The simulations using the refractive (Equation (1)), reflection, and reflection and transmission (Equation (2)) properties of air (gap between prism-to-prism contact surfaces) and BK7 glass allowed visualization and determination of the dimensions and angles of the 3-prism assembly. Note that the BK7 glass refraction coefficients varied slightly from 1.530 to 1.509 for 405 nm to 904 nm, respectively. As the aim of the 3CCD system was for use from 700 to 1000 nm, an average coefficient value for the NIR region, 1.510, was used for the simulation. Note that the refraction coefficient values at 786, 830, and 904 nm were 1.511, 1.510, and 1.509, respectively. Optimal dimensions of the three-prism assembly were determined through iterative trials to achieve the 35-mm back focal length and 6.66 × 5.32 mm diagonal focal plane size:

(1) $$\eta_{1}\sin\theta_{i}\equiv \eta_{2}\sin\theta_{t}$$

where *η* is refractive index for air =1.0 and for BK7 glass =1.510, and *θ_i_*, *θ_t_* are incident and transmission angles, respectively:

(2) $$r^{2}=\frac{{\left(\frac{\tan\left(\theta_{i}-\theta_{t}\right)}{\tan\left(\theta_{i}+\theta_{t}\right)}\right)}^{2}+{\left(\frac{\sin\left(\theta_{i}-\theta_{t}\right)}{\sin\left(\theta_{i}+\theta_{t}\right)}\right)}^{2}}{2},\;\text{and}\;r^{2}+t^{2}\frac{\eta_{2}\cos\theta_{t}}{\eta_{1}\cos\theta_{i}}=1$$

where *r* and *t* are reflection and transmission coefficients, respectively.

**Figure 3.**
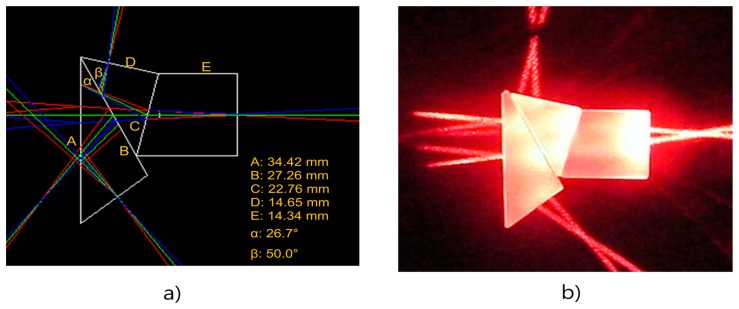
(**a**) Dimensions of the three-waveband beam splitter prisms determined by the simulation software; (**b**) Photo of the prism assembly manufactured with BK7 optical glass. Two-line laser were used to confirm the back focal lengths of the beam splitter assembly.

Figure 3a shows the optimal prism shapes and dimensions determined by the iterative computer simulations. The prism assembly manufactured with the BK7 optical glass is shown in Figure 3b. Two 670-nm line lasers were used to ensure the 35 mm back-focal length of the prism assembly, shown as the cross-section of two laser lines in Figure 3b.

### 3.2. Integration of Electronic Components

**Figure 4.**
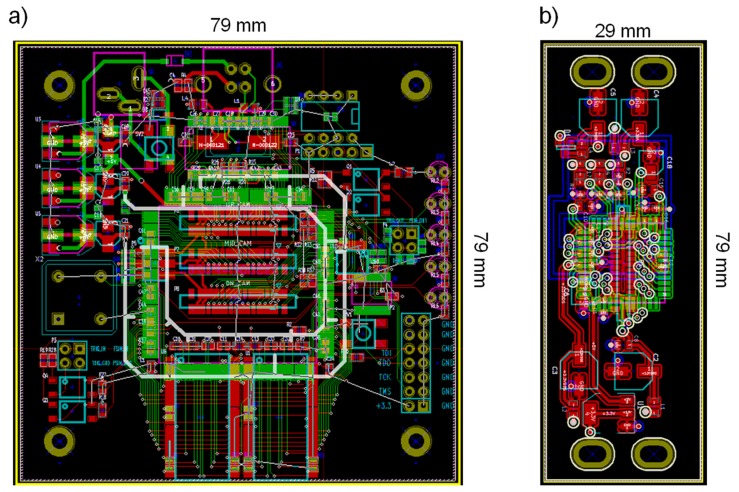
Gerber files for (**a**) main board containing FPGA, USB FIFO, two RAMs, and I2C multiplexer; and (**b**) CCD image sensor.

Key electrical components for simultaneous 3CCD image interface-data transfer and management included the FPGA, USB FIFO, RAM, and Inter-Integrated Circuit (I2C) multiplexer. The electronic components were integrated on a printed circuit board (PCB) designed in-house using open source suite for Electronic Design Automation KiCad. Figure 4 shows Gerber files for the main PCB (containing the FPGA, RAM, USB FIFO, and I2C multiplexer), and a PCB for a CCD sensor. Three CCD PCBs were built and each CCD image sensor mounted on a PCB with independent power supply circuit is connected to the main board by Flat Flexible Cable (FFC) with 150-mm length and 0.5-mm pin-pitch. The power (5V DC) is provided via the USB connection from the host computer. A voltage regulator (LM1117, Linear Technology, Milpitas, CA, USA) circuit was integrated on the main PCB to supply the necessary power to individual electric components.

Integrated electrical components on PCBs and firmware programming allowed parallel processing, synchronization, and independent control of the three CCD images without the loss of data and ensured the transfer of data without significant delay. On the FPGA module (main PCB), most of the interfaces except the USB communication are processed through firmware software written in Verilog hardware description language (HDL; ISE Design Suite 11.5, Xilinx). The USB transmission and CCD sensor control from/to host computer via I2C multiplexer are processed on USB FIFO programmed in C-language (uVision2 C51 Development Tool, Keil, Plano, TX, USA).

**Figure 5.**
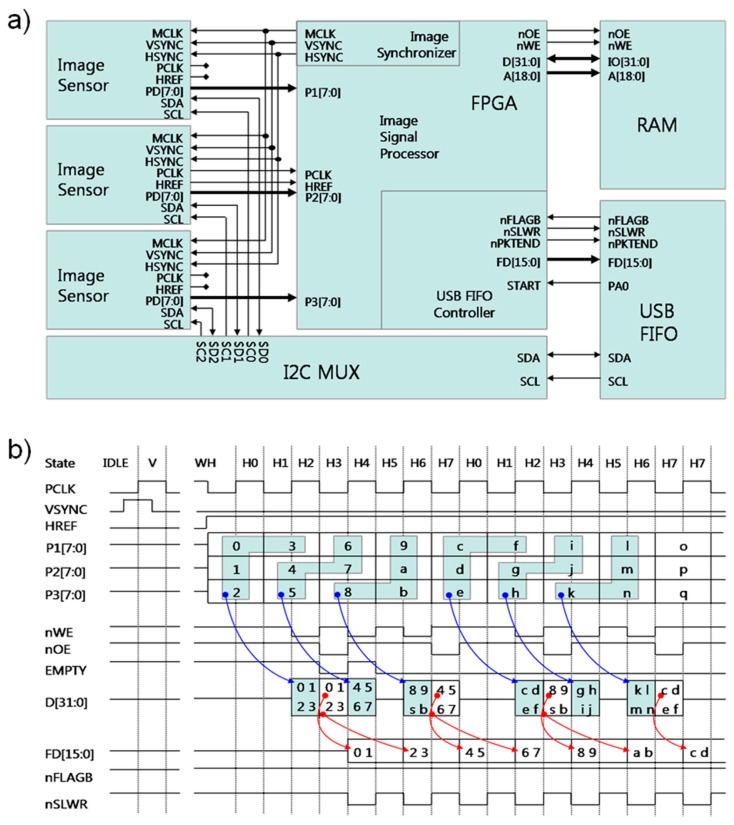
(**a**) Schematic view with 3CCD image sensors, FPGA, RAM, USB FIFO and I2C multiplexer; (**b**) Timing diagram of image signal processing, data buffering, and USB transferring.

The most significant feature of this newly developed 3CCD camera is its capability for parallel processing. The FPGA module takes a multifunctional role that makes image transmission of three image sensors identical, processes image data simultaneously, buffers 8-bit image data on the RAM module to mitigate the loss of pixel data prior to transmitting data through the USB FIFO; the FPGA device has software blocks including image synchronizer, image signal processor (parallel processing), and USB FIFO controller (Figure 5a). The image synchronizer generates operating clocks (MCLK), vertical sync signals (VSYNC), and horizontal sync signals (HSYNC) in order to ensure that the CCD sensors transfer identical pixel clock (PCLK) and horizontal reference signal (HREF). In these synchronized pixel data (P1[7:0], P2[7:0], and P3[7:0]), the image signal processor does parallel processing to save all the pixel data instantly as described in Figure 5b; pixel data, being transferred simultaneously, are registered on 32-bit data (D[31:0]) via inter-pixel data pairing (blue arrow), and then, that is temporally buffered on RAM (two-512 Kbyte with 16 bits data width) through memory write operation being assertion of low level nWE pin.

In normal operation, the USB FIFO is available (nFLAGB pin with high level), the USB FIFO controller brings back buffered pixel data on D[31:0] register, and then independently transfers 32-bit data over 16-bits register (FD[15:0]) twice as the nSLWR pin (red arrow) turns to low-level. In these ways, FPGA simultaneously transfers all the image data, without data loss, and significant delay, into the USB FIFO device. On the front end of the USB communication, USB FIFO device actually transfers pixel data under Bulk-mode protocol with transfer guarantee, and is able to individually control each CCD parameters, including gain, brightness, and shutter speed, with help of I2C multiplexer.

### 3.3. Housing Unit

**Figure 6.**
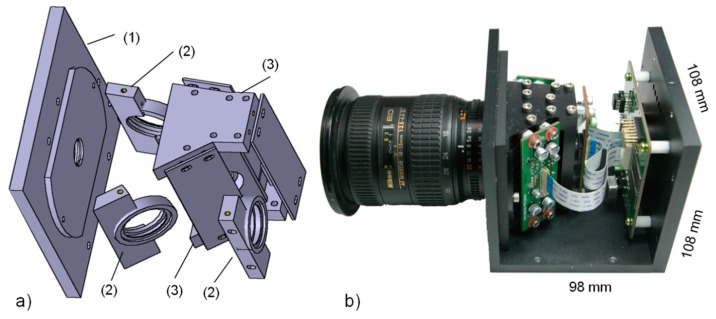
(**a**) 3D illustration of internal frame/structure of the portable 3CCD camera system: (1) F-mount lens holder; (2) interference filter holders; and (3) prism assembly cover. (**b**) Photo of the portable 3CCD camera system showing fully assembled internal components.

The camera housing unit and frames for internal components were constructed with anodized aluminum. Figure 6a shows schematic illustrations of the frames for mounting an object lens, the image beam splitter assembly, the interference filters/holders, and the CCD image sensors with the PCB. The top and bottom covers retain the beam-splitter assembly in place. The interference filter (25 mm) holders are placed between the beam splitter and the CCD modules, and are removable—the interference filters can be changed without disrupting the focal plane/alignments of the optics and CCD sensors. The lens holder has an F-mount flange for use with a Nikon lens (e.g., AF-Nikon f35-75 mm F/3.3-4.5, Nikon, Tokyo, Japan).

Figure 6b shows the inside view of the fully assembled 3CCD camera system with the placement of the main PCB containing the FPGA, USB FIFO, RAMs, and I2C multiplexer. Three CCD-PCB modules are connected to the main PCB via flat flexible cables. The external dimensions of the frame are 108 × 98 × 108 mm.

### 3.4. Software Interface

The USB driver and interface/application software for operation on a Windows-based PC was written in Visual C++. The software displays the live-streaming multispectral images, and adjusts independent imaging parameters for individual CCD sensors such as gain and exposure time. Independent imaging parameters are controlled from the host computer via I2C Multiplexer in 8-bit levels. The 3CCD camera system was initially designed to continuously stream 30 frames of 24-bit image data per second where each 24-bit image data contains three 8-bit multispectral images. Thus, the longest exposure time allowed for streaming images was limited to 20 ms.

### 3.5. Multispectral Apple Images

**Figure 7.**
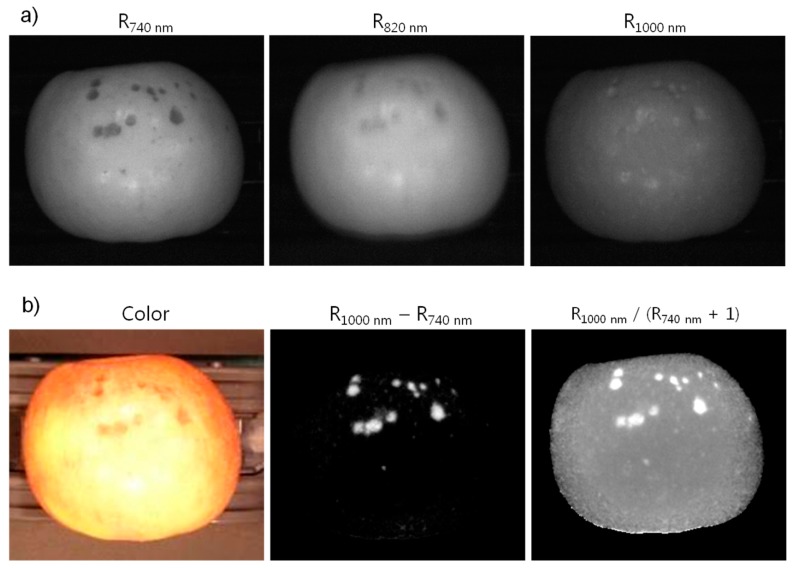
(**a**) Multispectral images of a Fuji apple acquired with the portable 3CCD imaging system. The images were acquired at the full pixel resolution (1280 × 1024); (**b**) Color photo of the Fuji apple, two-band difference image, and two-band ratio image. The difference and ratio images highlight the defect regions of the apple.

Multispectral images at 740, 820, and 1000 nm acquired with the portable 3CCD camera for Fuji apples with defects are shown in Figure 7a. The FWHM (full width at half maximum) of the interference filters was 10 nm. The images were acquired with 20 ms exposure time for each CCD sensor. However, the gain for each individual CCD sensor was adjusted to result in three images with approximately equivalent brightness or gray-scale responses. The illumination was provided using a hemispherical dome light consisting of eight 150-W incandescent lamps with adjustable intensity. Apples surfaces are specular and the circular arrangement of the eight lamps for illuminating the samples is observable in the multispectral images.

Several investigations have reported advantages of using the NIR portion of the spectral region for detection of defects on apples since the responses are insensitive to color variations of the apple surfaces. In addition, simple two-band algorithms, such as difference and ratio imaging, have been shown to be effective in identification and detection of defect spots on the surfaces of apples [4,9,23]. Note that none of the studies utilized the 1000 nm waveband for detection of defects. However, we employed the 1000 nm band to demonstrate imaging in the extreme spectral response edge of the silicon-based CCD sensors. The representative sample images along with the difference and ratio images in Figure 7b clearly highlighting the defect regions on the apple surfaces demonstrate the potential utility of the presented 3CCD system.

## 4. Conclusions

RGB-based color imaging devices are the predominant tools in machine vision applications for many industries. For agro-food industries, color imaging provides assessments of quality attributes such as color, size, and shape of agricultural commodities. However, recent studies have suggested the need for imaging devices capable of multispectral imaging beyond the color region to enable other quality and safety evaluations for agricultural commodities. Conventional multispectral imaging devices lack flexibility in spectral waveband selectivity for such applications. The 3-waveband beam-splitter prism assembly in this study was designed to accommodate the placement of interference filters instead of the dichroic coating more typically used on prisms, and to further allow changing of the filters for application-specific multispectral imaging. We designed and integrated key electrical components on PCBs and firmware programming that allows for parallel processing, synchronization, and independent control of the three CCD images without loss of data and ensures transfer of data without significant delay. The utility of the developed 3CCD was illustrated with multispectral imaging in the NIR for detection of defects on apples. We envision that the presented 3CCD can be used in a number of other research and industry applications.
